# Predictors of poor precautionary practices towards COVID-19 among cancer patients

**DOI:** 10.2217/fon-2021-0193

**Published:** 2021-09-02

**Authors:** Abdul R Jazieh, Assia A Bensalem, Adda Bounedjar, Zineb Benbrahim, Mohamed O Alorabi, Atlal M Abusanad, Emad M Tashkandi, Muath AMA Alnassar, Abdul RAD El Kinge, Sana Al-Sukhun, Abdullah Alsharm, Hassan Errihani, Nafisa A Abdelhafiez, Mohammad Alkaiyat, Hoda Jradi

**Affiliations:** ^1^Cincinnati Cancer Advisors, Cincinnati, OH, USA; ^2^Medical Oncology Department, Établissement Hospitalier DIDOUCHE Mourad, Faculté de medicine, Université de Constantine 3, Constantine, Algeria; ^3^Université Blida 1 Laboratoire de cancérologie, Faculté de Médecine, BP 270, Route de soumaa, Blida, Algeria; ^4^Medical Oncology, Faculty of Medicine & Pharmacy of Fez, University Sidi Mohamed Ben Abdellah, Fez, Morocco; ^5^Ain Shams Faculty of Medicine, Clinical Oncology Department, Cairo, Egypt; ^6^Faculty of Medicine, King Abdulaziz University, Jeddah, Saudi Arabia; ^7^College of Medicine, Umm Alqura University, Oncology Center, King Abdullah Medical City, Makkah, Saudi Arabia; ^8^Kuwait Cancer Control Cancer, Kuwait City, Kuwait; ^9^Hematology Oncology Service, NMC Royal Hospital Sharjah, Sharjah University, Sharjah, UAE; ^10^Hematology/Medical Oncology, Al-Hyatt Oncology Practice, Amman, Jordan; ^11^Comprehensive Cancer Center, King Fahad Medical City, Riyadh, Saudi Arabia; ^12^National Institute of Oncology, Mohammed V University, Rabat, Morocco; ^13^Department of Oncology, King Abdulaziz Medical City, MNGHA, Riyadh, Saudi Arabia; ^14^Department of Oncology, King Abdulaziz Medical City, King Saud bin Abdulaziz University for Health Sciences, King Abdullah International Medical Research Center, Riyadh, Saudi Arabia; ^15^College of Public Health & Health Informatics, King Saud Bin Abdulaziz University for Health Sciences, King Abdullah International Medical Research Center, Riyadh, Saudi Arabia

**Keywords:** behaviors, cancer patients, coronavirus, COVID-19, pandemic, precautions

## Abstract

**Objective:** Our study goal was to evaluate the behavioral response and practices of cancer patients to the coronavirus disease 2019 (COVID-19) pandemic in the Middle East and north Africa. **Methods:** A cross-sectional study was conducted using a validated anonymous 45-question survey administered via SurveyMonkey^®^ to cancer patients in 13 centers in Algeria, Egypt, Jordan, Kuwait, Morocco and Saudi Arabia. **Results:** During the study period (from 21 April to 30 May 2020), 3642 patients participated in the study. The majority of patients (84.81%) were worried about contracting the infection. The reported strict adherence to precautions included avoiding the following actions: hand-shaking (77.40%), hugging and kissing (82.89%), social gathering (90.09%), meeting friends (84.68%) and visiting markets (75.65%). In a multivariate analysis, patients with poor precautionary practices were about twice as likely to cancel their medical appointment or a treatment session. **Conclusion:** Improving cancer patients’ knowledge of and adherence to precautionary measures is needed not just to reduce the risk of acquiring infection but also to minimize the interruption of their medical care.

## Lay abstract

COVID-19 poses a higher risk for patients with cancer than other patients; therefore, it is prudent that they adhere to precautionary measures to protect themselves from the infection. We conducted a study to evaluate the behaviors and practices of these patients in response to the COVID-19 pandemic in the Middle, East and North Africa. We developed a survey of 45 questions that was distributed in 13 centers in Algeria, Egypt, Jordan, Kuwait, Morocco and Saudi Arabia between 21 April and 30 May 2020. About 85% of the 3642 patients who participated in the study were worried about contracting the infection. A substantial percentage of them (10–30%) were not adhering to various precautions and social distancing rules. On the other hand, 16% of them canceled medical appointments and 12% canceled treatment sessions. Our study showed the need for better adherence of patients with cancer to the infection precautions and most importantly, the need to have a better compliance with their treatment plans, such as keeping their scheduled appointments, to avoid harms from treatment delays.

The pandemic of coronavirus disease 2019 (COVID-19) had major devastating effects on a global level due to its associated morbidity and mortality and the severe disruption of healthcare delivery in many countries. By 19 June 2021, more than 177 million people had contracted the infection, with more than 3.8 million deaths worldwide [1]. Although the risk of a worse outcome is higher in certain populations, especially the elderly and people with comorbidities, the disease affects people of all ages and backgrounds.

Cancer patients are a vulnerable population for many reasons, including being immunocompromised from the cancer or its treatment, being generally older with other comorbidities and risk factors such as smoking, in addition to being frequent visitors to healthcare facilities for various procedures and interventions. Therefore they are at a high risk of contracting infections and developing serious complications from these infections. Furthermore, interruptions in cancer care have been encountered during the pandemic due to providers’ decisions, patients’ concerns or overwhelmed healthcare systems. However, the response of oncology providers and practices to the pandemic may have contributed to misconceptions and concerns among patients about the risks of the pandemic, especially in its early phases. Interventions such as closing cancer care facilities (partially or totally), canceling patients’ appointments, delaying treatment, switching to oral therapy or longer interval therapies, postponing elective testing or procedures (including surveillance of survivors), providing care near home (such as laboratory testing) would certainly impact the patients’ perceptions and behaviors [2–7].

For these reasons, patients with cancer are expected to be aware and vigilant about behavioral changes and lifestyle modifications that minimize the risk of contracting SARS-CoV-2 infection and to ensure their safety. These precautions include improving their personal hygiene, wearing masks and paying more attention to new emerging concepts such as social distancing and self-isolation. On the other hand, avoiding their visits to healthcare facilities during this pandemic may impact the continuity of cancer care, leading potentially to a poor cancer-related outcome.

The pandemic was associated with a flood of information that was overwhelming due to its sheer volume. In addition, ‘fake news’ and information coming from untrustworthy sources go viral in this era of social media and communication platforms [8].

Although there are studies reporting knowledge, attitudes and practices in the Middle East and North Africa (MENA), no studies have yet investigated these issues among cancer patients [9,10]. Therefore it is critical to assess the common beliefs, misconceptions and behaviors that are prevalent among cancer patients to understand how they are reacting to the pandemic. Knowing the behaviors and concerns of patients will enable oncologists and other healthcare providers to address these issues in more effective ways [11,12].

This study aims to evaluate these issues among cancer patients in the MENA region and to gauge changes in their behaviors in response to the COVID-19 pandemic and their approaches to staying safe.

## Patients & methods

This cross-sectional study was conducted on a sample of cancer patients from hospitals in Saudi Arabia, Algeria, Morocco, Kuwait, Egypt and Jordan to determine the level of knowledge and level of preventive behavior and their associated factors.

All cancer patients who agreed to participate in the study were included, with no exclusion criteria except declining to participate. Subsite investigators were involved from the participating centers. Electronic and paper-based versions of the survey were distributed. The electronic survey was sent via text messages to patients. Paper-based versions were printed and filled in by patients and then staff computed the data. Patients’ consent was obtained for both methods. All questions were made mandatory to avoid missing values.

The study was approved first by King Abdullah International Medical Research Center at Ministry of National Guards Health Affairs (IRB NO: RC20\144\R) and all other collaborators obtained relevant institutional review board approval from their institutions.

### Study instrument

A validated electronic questionnaire built on SurveyMonkey^®^ (www.surveymonkey.com) was used for data collection. The instrument was distributed by the subsite investigators in the participating centers. Subsites used the same link to the instrument to collect the data uniformly from all patients.

The instrument was constructed based on a preliminary review of the literature reporting on patients’ knowledge, perception and practices regarding infectious diseases. Further development of the questionnaire included interviewing patients regarding their recent healthcare-seeking experiences during the COVID-19 outbreak. Information collected from the literature and the emerging concepts from the initial patients’ interviews guided the development of the questionnaire. Development in the Arabic language and the content validity of the questionnaire was confirmed by seeking the appraisal of five experts (oncologists), and a content validity index was calculated. The face validity of the questionnaire was evaluated by administering it to a group of ten patients to ensure that all terms used were understandable and to clarify any ambiguity. The modified final questionnaire was piloted on a small group of patients (n = 25) for a final check and assessment of clarity, time management and consistency. Reliability was tested by reporting internal consistency and test–retest reliability. Test–retest reliability was conducted by readministering the questionnaire to the same group of patients (n = 20), and the Pearson correlation coefficient was calculated for the developed tool. The correlation for the two administrations was 0.82, indicative of adequate test–retest reliability. Internal consistency was also adequate for knowledge and practice constructs (Cronbach’s coefficient α of 0.78 and 0.77).

The first section of the study instrument included sociodemographic information such as age, level of education, occupation, marital status, type of disease, type of treatment and the last date of received treatment. The second section included general knowledge questions regarding possible transmission modes and signs and symptoms of COVID-19. Additionally, statements were included that reflected the patient’s perceived risk of contracting COVID-19, behavioral modifications, preventive behaviors related to wearing protective gear and social distancing, and perceived effectiveness of preventive behaviors. The knowledge questions were scored according to ‘yes’, ‘no’ or ‘uncertain’. Preventive behaviors were assessed using a four-point scale (1, never; 2, sometimes [two or three times a day]; 3, often [five times a day]; 4, always). Participants also provided information on their disease management during the pandemic, appointment and treatment cancellation, whether they were worried about contracting the infection, and about their preferred sources and channels of information.

Levels of knowledge on infection and transmission of COVID-19 and levels of preventive behavior among cancer patients were assessed based on their responses to questions seeking the information. A composite variable for knowledge and preventive behaviors was constructed based on the final score calculated by coding the correct answers to knowledge questions and adequate preventive behavior as 1 and adding up all responses to obtain the final scores. Participants who scored 6 or less were considered to have a poor knowledge level, while participants who scored more than 6 were classified as having a good level of knowledge. Similarly, participants who scored 3 or less were considered to have poor preventive practices and those who scored more than 3 were considered to practice good preventive behavior toward COVID-19. The composite variables for level of knowledge and level of preventive behaviors were reliable (Cronbach’s α = 0.64 and 0.73, respectively) in representing knowledge and preventive behavior factors in the analysis.

### Data analysis

All responses were collected anonymously, and once the data collection process was completed, data were imported from the Excel sheets into Stata (version 14.0; StataCorp, LLC, TX, USA) for analysis. Study variables were summarized, in aggregate, using standard descriptive statistics such as mean, standard deviation, frequency and proportions. Pearson’s χ-squared test of independence and p-values were used to assess any differences across the patients’ characteristics by level of preventive behavior from COVID-19. To determine associated factors for poor preventive behavior toward SARS-CoV-2 infection, odds ratios and their respective 95% CIs were computed using multivariate logistic regression analysis. All variables that showed significance in the bivariate analysis were entered into the multivariate analysis. The Hosmer and Lemeshow goodness-of-fit test was used to assess the calibration of the model. The statistical significance was based on a p-value of ≤ 0.05.

### Patient & public involvement

As mentioned above, a cohort of patients were involved in content validation a test and retest of reliability. Once the study is published, we will share the results with our patients and publicize it.

## Results

Between 21 April and 30 May 2020, a total of 3642 patients were enrolled in the study. The patients’ median age was 55 years (range: 14–97), 59.23% were female and 75.37% reported being married. Breast cancer was the most common malignancy reported and 83% had received cancer therapy in the last 3 months. Table 1 shows the patients’ characteristics.

**Table 1.** Patient characteristics (n = 3642).

Characteristics	n	%
Age (years); median = 55; range = 14–97: – ≤39 – 40–49 – 50–59 – 60–69 – ≥70	558 729 918 770 699	15.19 19.84 24.99 20.96 19.03
Sex: – Male – Female	1485 2175	40.77 59.23
Education: – Illiterate – Primary – Middle – High school – College – Higher degree	616 637 817 775 698 99	16.91 17.49 22.43 21.28 19.17 2.72
Marital status: – Single – Married – Divorced – Widowed	345 2745 323 229	9.47 75.37 8.87 6.29
Country: – Algeria – Saudi Arabia – Morocco – Jordan – Egypt – Kuwait	2659 440 288 42 143 70	73.01 12.08 7.91 1.15 3.93 1.92
Cancer type: – Breast – Gastrointestinal – Lung cancer – Genitourinary – Gynecological – Head and neck – Others – Unknown	1123 878 348 278 153 124 320 418	30.83 24.11 9.56 7.63 4.20 3.40 8.79 11.48
Disease status: – Disease-free – Active disease under control – Uncontrolled disease – Do not know	788 2102 335 417	21.64 57.72 9.20 11.45
Last treatment date: – <15 days – 15 days to <30 days – 30 days to 3 months – >3 months to 6 months – >6 months	758 1408 719 286 471	20.81 38.66 19.74 7.85 12.93
Treatment received in the last 6 months: – Surgery – Radiotherapy – Chemotherapy – Biology (non-cytotoxic cancer therapy) – No treatment	1016 87 2605 531 397	27.90 23.81 71.53 14.58 31.26
Current treatment: – Intravenous – Oral – None	2909 920 282	79.87 25.26 7.74

## Patients’ knowledge

The majority of participants knew about the symptoms of COVID-19, especially fever and cough and, to a lesser extent, fatigue. More than one-third of the participants either did not believe or did not know that an asymptomatic person can be a carrier of the virus. About three-quarters of patients did believe that infection is more severe in immunocompromised patients. A fraction of the participants either did not believe or did not know about the protective value of mask wearing, hand washing and using sanitizers. A majority of the patients reported being either very worried (46.81%) or slightly worried (38%) about contracting the infection (Table 2).

**Table 2.** Patients’ knowledge about COVID-19.

Knowledge items (mean = 6.06; standard deviation = 1.90)	n	%
Symptoms related to COVID-19: – Fatigue – Fever – Dry cough	2086 3124 2947	57.28 85.78 80.29
An infected person with no symptoms can be a carrier of COVID-19: – Yes – No – Do not know	2283 354 1005	62.69 9.72 27.59
COVID-19 is more severe in immune-compromised patients?: – Yes – No – Do not know	2793 181 668	76.69 4.97 18.34
Appropriate distance to be kept between individuals (meters): – <1 – 1–1.5 – >1.5	217 2616 321	6.88 82.94 10.18
Wearing a mask is protective: – Yes – No – Do not know	2518 317 807	69.14 8.70 22.16
Washing hands is protective: – Yes – No – Do not know	2916 129 597	80.07 3.54 16.39
Using sanitizer is protective: – Yes – No – Do not know	2591 241 810	71.14 6.62 22.24
Worried about contracting infection: – Very worried – Slightly worried – Not worried at all	1705 1384 553	46.81 38.0 15.18

## Patients’ behaviors

Although the majority of patients reported using precautionary measures such as repeated hand washing, using sanitizers and wearing masks, a substantial number of them did not take these precautions. Furthermore, a significant number of participants did not observe social distancing measures, as they reported visiting crowded areas, shaking hands, hugging others and not keeping distance from others (Table 3).

**Table 3.** Patients’ precautionary practice during COVID-19 pandemic (n = 3642).

Precautionary behavior items	n	%
Repeated hand washing: – Always – Sometimes/often – Never	2459 786 397	67.52 21.58 10.90
Wearing masks in public areas: – Always – Sometimes/often – Never	2581 733 328	70.87 20.31 9.01
Attendance of large social events: – Yes – No	361 3281	9.91 90.09
Meetings with friends: – Always – Sometimes/often – Never	104 454 3084	2.86 12.47 84.68
Shaking hands with others: – Always – Sometimes/often – Never	206 617 2819	5.66 16.94 77.40
Hugging and kissing others: – Always – Sometimes/often – Never	115 508 3019	3.16 13.95 82.89
Visiting malls and shopping centers: – Always – Sometimes/often – Never	97 790 2755	2.66 21.69 75.65
Keeping safe distance from others: – Always – Sometimes/often – Never	2146 1008 488	58.92 27.68 13.40

mean = 3.03; standard deviation = ± 2.03; range = 0–11.

### Medical care & alternative measures

Almost 21% of patients admitted that they would not attend a previously scheduled medical appointment at this time, and others canceled their appointments (16%) or treatment sessions (12.66%). The majority preferred a virtual doctor visit over an in-person visit. Table 4 depicts the association between disease management choices and participants’ characteristics.

**Table 4.** Association between participants’ characteristics and management practices during the COVID-19 pandemic.

Variable	Showing up for medical appointment, OR (95% CI)	Calling medical team for respiratory symptoms, OR (95% CI)	Canceling appointment by patient request, OR (95% CI)	Canceling treatment by patient request, OR (95% CI)	Prefer virtual appointment, OR (95% CI)
Male gender	1.39 (1.18–1.65)	0.76 (0.66–0.88)	0.98 (0.82–1.18)^†^	1.32 (1.08–1.61)	1.08 (0.89–1.33)^†^
Married	0.68 (0.53–0.87)	1.24 (1.01–1.51)	0.89 (0.84–0.95)	0.88 (0.83–0.94)	1.30 (1.00–1.69)
Good knowledge	0.60 (0.49–0.73)	1.03 (0.84–1.31)^†^	1.49 (1.20–1.85)	1.08 (0.87–1.36)^†^	1.50 (1.22–1.84)
Good precautions	0.55 (0.47–0.65)	1.64 (1.43–1.89)	0.40 (0.32–0.49)	0.25 (0.19–0.32)	1.01 (0.96–1.07)^†^
Disease under control	0.24 (.17–0.34)	1.04 (0.83–1.31)^†^	1.169 (0.86–1.57)^†^	1.67 (1.17–2.39)	1.49 (1.12–1.97)
Disease free	0.64 (0.47–0.89)	1.32 (1.03–1.71)	1.80 (1.13–2.27)	5.23 (3.43–7.96)	1.84 (1.31–2.57)
Treatment (chemotherapy/surgery) within last 6 months	3.98 (3.35–4.73)	1.15 (0.97–1.35)^†^	1.78 (1.35–2.36)	5.62 (3.75–8.44)	0.61 (0.46–0.79)
Worried about COVID-19	0.68 (0.58–0.80)	1.43 (1.24-.64)	1.42 (1.18–1.71)	1.92 (1.57–2.35)	3.20 (2.56–4.00)

† Not significant.

OR: Odds ratio.

### Predictors of poor precautionary practices

There were multiple variables associated with poor precautionary practices among participants, as depicted in Table 5. However, a multivariate analysis revealed that the most significant predictors of poor precautionary practices include male gender, educational level lower than college, being widowed or divorced, being disease free or having unknown disease status, and receiving surgery (Table 6). Unlike receiving surgery, receiving chemotherapy was inversely associated with poor precautionary practices.

**Table 5.** Descriptive data of study sample characteristics by history of practicing precautionary behaviors during the COVID-19 pandemic.

Characteristic	Level of precautionary behaviors, n (%)			p-value
	All (n = 3642), n (%)	Good (n = 1537; 42.20%)	Poor (n = 2105; 58.68%)	
Age (years): – ≤29 – 30–39 – 40–49 – 50–59 – 60–69 – ≥70	158 (4.30) 400 (10.89) 729 (19.84) 918 (24.99) 770 (20.96) 699 (19.03)	76 (48.10) 179 (44.75) 362 (49.66) 433 (47.17) 287 (37.27) 200 (28.61)	82 (51.90) 221 (55.25) 367 (50.34) 485 (52.83) 483 (62.73) 499 (71.39)	< 0.001
Sex: – Male – Female	1485 (40.77) 2375 (59.23)	539 (36.30) 946 (39.83)	998 (67.20) 1159 (48.80)	< 0.001
Education: – Illiterate – Primary – Middle – High school – College or more	616 (16.91) 637 (17.49) 817 (22.43) 775 (21.28) 797 (21.88)	227 (36.85) 248 ((38.93) 336 41.12) 323 (41.68) 403 (50.56)	389 (63.15) 389 (61.07) 481 (58.87) 452 ((58.32) 394 (49.43)	< 0.001
Marital status: – Single – Married – Divorced – Widowed	345 (9.47) 2745 (75.37) 323 (8.87) 229 (6.29)	161 46.67) 1249 (45.50) 64 (19.81) 63 (27.51)	184 (53.33) 1496 (54.50) 259 (80.19) 166 (72.49)	< 0.001
Country: – Algeria – Saudi Arabia – Morocco – Jordan – Egypt – Kuwait	2659 (73.01) 440 (12.08) 288 (7.91) 42 (1.15) 143 (3.93) 70 (1.92)	1056 (39.71) 202 (45.90) 180 (62.50) 30 (71.43) 32 (22.38) 37 (52.86)	1603 (60.29) 238 (54.09) 108 (37.50) 12 (28.57) 111 (77.62) 33 (47.14)	< 0.001
Disease status: – Disease-free – Active disease under control – Uncontrolled disease – Do not know	788 (21.64) 2102 (57.72) 335 (9.20) 417 (11.45)	318 (40.35) 950 (45.19) 84 (25.07) 185 (44.36)	470 (59.64) 1152 (54.80) 251 74.92) 232 (55.64	< 0.001
Disease type: – Breast – Gastrointestinal – Lung – Genitourinary – Gynecological – Head and neck – Others – Unknown	1123 (30.83) 878 (24.11) 348 (9.56) 278 (7.63) 153 (4.20) 124 (3.40) 320 (8.79) 418 (11.48)	534 (47.55) 348 (39.64) 70 (25.18) 134 (38.51) 64 (41.83) 46 (37.10) 131 (40.94) 210 (50.24)	589 (52.45) 530 (60.36) 208 (74.82) 214 (61.49) 89 (58.17) 78 (62.90) 189 (59.06) 208 (49.76)	< 0.001
Treatment in the last 6 months: – Surgery – Chemotherapy	1016 (27.90) 2605 (71.53)	342 (33.66) 1032 (39.62)	674 (66.34) 1573 (60.38)	< 0.001
Worried about contracting infection: – Very worried – Slightly worried – Not worried at all	1705 (46.81) 1384 (38.00) 553 (15.18)	853 (50.03) 493 (35.62) 191 (34.54)	852 (49.97) 891 (64.38) 362 (65.46)	< 0.001
Disease management practices				
Showing up to medical appointment: – Yes – No	2901 (79.65) 741 (20.35)	1138 (39.23)	1763 (60.77)	< 0.001
Canceling medical appointment: – Yes, per patient request – Yes, per medical team advice – No	584 (16.04) 935 (25.67) 2123 (44.56)	147 (25.17) 416 (44.49) 974 (45.88)	437 (74.83) 519 (55.51) 1149 (54.12)	< 0.001
Canceling treatment session: – Yes, per patient request – Yes, per medical team request – No	461 (12.66) 602 (16.53) 2579 (70.81)	80 (17.35) 279 (46.34) 1178 (45.68)	381 (82.65) 323 (53.65) 1401 (54.32)	< 0.001
Source of information for COVID-19				
Internet	1461 (40.12	682 (46.68)	779 (53.31)	< 0.001
Television	3117 (85.58)	1408 (45.17)	1709 (54.3)	< 0.001
Family and friends	1433 (39.35)	540 (37.68)	893 (62.32)	< 0.001
Social media	1551 (42.22)	709 (45.71)	842 (54.29)	< 0.001
Knowledge regarding COVID-19: – Good – Poor	2641 (71.88) 1033 (28.12)	1217 (46.08) 320 (30.98)	1424 (53.92) 713 (69.02)	< 0.001

**Table 6.** Results of multivariate logistic regression analysis for factors associated with poor practice of precautions toward COVID-19.

Characteristic	Reference	OR	Wald	p-value	95% CI
Male	Female	1.40	3.95	< 0.001	1.18–1.65
Illiterate Primary education Middle school education High school education	College or more	1.80 1.81 1.45 1.45	4.02 4.21 2.87 2.45	< 0.001 < 0.001 0.004 0.014	1.35–2.40 1.37–2.39 1.12–1.87 1.06–1.71
Divorced/widowed	Married	2.10	6.02	< 0.001	1.65–2.68
Disease free Uncontrolled disease Do not know	Active disease under control	1.74 0.77 2.33	5.04 -2.06 5.44	< 0.001 0.039 < 0.001	1.40–2.16 0.61–0.99 1.72–3.17
Genitourinary cancer Other rare types of cancer	Breast cancer	1.87 0.65	3.49 -3.30	< 0.001 0.001	1.31–2.66 0.51–0.84
Treatment with surgery	No treatment	1.29	3.31	< 0.001	1.11–1.51
Treatment with chemotherapy	No treatment	0.79	-3.08	0.002	0.67–0.91
Slightly/not worried about infection	Worried	1.69	6.15	< 0.001	1.41–1.95
Showing up to medical appointment	No show	1.70	4.88	< 0.001	1.35–2.03
Take symptoms medications Go to local clinic for symptoms Go to hospital for symptoms	Call medical team	1.47 1.31 1.42	2.51 2.55 3.57	0.012 0.011 < 0.001	1.09–1.98 1.07–1.62 1.17–1.73
Canceling medical appointment per patient request	No cancellation	1.75	4.12	< 0.001	1.34–2.29
Canceling treatment session per patient request	No cancellation	2.24	4.92	< 0.001	1.63–3.09
Television as source of information Family and friends as source of information	Other	0.48 1.52	-5.77 4.84	< 0.001 < 0.001	0.37–0.61 1.28–1.81
Poor knowledge regarding COVID-19	Good knowledge	1.82	6.44	< 0.001	1.52–2.18

OR: Odds ratio.

## Discussion

Our study included many patients from different countries and different socioeconomic and educational backgrounds, which provided us with an adequate sample size to conduct multiple analyses and explore many variables. The majority of patients were older than 50 years, and breast cancer was the most common diagnosis. Two-thirds of patients were either actively on treatment or had completed treatment within 3 months of the survey administration, with chemotherapy being the most frequently reported treatment modality, particularly intravenous chemotherapy. More than two-thirds were worried about contracting COVID-19. Our study cohort showed a good knowledge of the precautionary procedures to protect against the infection. Knowledge about hand hygiene and wearing masks was translated to adequate safety practices, except for keeping a safe distance. With the emergence of the pandemic, hand hygiene was the most advertised protective measure, which may have steered attention away from the need for safe distancing. Another possible explanation is that some individuals may think that hand hygiene and wearing masks are alternatives to physical distancing. However, the reason for this observation is not entirely clear, and adherence to each safety measure must be encouraged, stressing that applying one measure instead of another is not a correct practice. Public health campaigns have to educate the masses that each measure has its own protective value, which is not interchangeable with the others [13]. The correlation between knowledge of COVID-19 and behaviors has been described in other studies. In a Chinese study of 6910 residents, multiple logistic regression analyses revealed that COVID-19 knowledge score (odds ratio: 0.75–0.90, p < 0.001) was significantly associated with a lower likelihood of risky attitudes and preventive practices toward the disease [14].

The published data of adherence to precautionary measures from general populations are variable. Our patients’ results are similar to the general population results published from Saudi Arabia [9,10,15,16]. Nonetheless, our patients should have shown stricter adherence; we expected that having a cancer diagnosis would act as a spur to taking better precautions [17].

Our study highlighted the importance of patients’ knowledge in determining many behaviors. Knowledge level was a significant predictor of whether the patient would choose not to show up for a medical appointment, to cancel the appointment and to prefer virtual clinic visits. Poor knowledge was a predictor of poor precautionary practice on univariate and multivariate analyses. Patients with cancer are in general very observant of their appointments and treatment sessions to ensure best outcomes; however, many of our participants expressed the intent to miss or cancel their appointments. Patients who reported being disease free and having the disease under control were more likely to express their intention not to attend medical appointments. This may be explained by their attempt to balance the potential risks of visiting the hospital against their disease status, which did not represent an immediate threat, unlike the potential exposure to the SARS-CoV-2 infection while visiting hospitals. On the other hand, patients receiving treatments within the last 6 months were more likely to attend appointments. Additionally, patients who exhibited good knowledge and executed good precautionary practices were more likely to avoid hospital visitations than the poor adherents. Tools to assist them in making a proper assessment of their disease status are essential; for instance, education regarding the ‘red flag’ symptoms that may need urgent intervention and providing alternative channels of communication in the case of uncertainty. Virtual clinic visits are desired, as the majority in this cohort expressed. Integrating telecommunications to allow timely access to cancer care and establishing the required infrastructures should be a priority for health authorities. Our study highlighted the importance of patients’ knowledge in determining many behaviors. Knowledge level was also a significant predictor of whether the patient would be choosing not to attend a medical appointment, canceling the appointment and preferring a virtual clinic visit.

As expected, most of the patients were either very worried or slightly worried about contracting the infection. This was also reported in a small sample of young adults with cancer, who were more concerned about catching the virus than their healthy peers [18,19]. A study of 630 adults from the USA’s general population showed that the percentage of individuals who were very worried about acquiring the disease was lower than the proportion of our cancer patients with a similar level of worry (24.6 vs 46.81%). However, the proportion of those who were not worried at all was close to that reported in our study (12.9 vs 15.18%). Having a cancer diagnosis might have contributed to the added level of worry and anxiety when compared with healthy individuals [16]. Our study showed that patients who reported being not worried or slightly worried are more likely to have poor precautionary practice than those who are very worried. Worry may reflect the perceived risk, which in turn was reported to increase implementation of protective behaviors [20].

Poor knowledge was a predictor of poor precautionary practice on both univariate and multivariate analyses. Moreover, predictors of poor precautionary practice in our study included male gender, low educational level, being widowed or divorced and poor knowledge of COVID-19. Patients who had been treated with surgery were more likely to have poor precautionary practice, while those receiving chemotherapy were less likely to have poor precautionary practice; this may reflect their perception of their susceptibility to contracting the infection. A noteworthy observation is that a lower educational level than college is associated with poor precautionary practices; the lower the level of education, the higher the likelihood of poor safety practices. Health authorities have to diversify education and communication means and simplify recommendations to achieve better dissemination of knowledge throughout all societal layers. Other studies have shown that race, poorer health and health literacy, low socioeconomic status, marital status and employments influenced the preparedness for a crisis [16].

The concerns regarding canceling patients’ appointments and treatment, irrespective of healthcare providers or patients instigating the cancellation, should be addressed. Guidelines and recommendations by various international entities were published to guide physicians on how to prioritize patient care [21–23]. In addition, physicians and healthcare systems should avoid the distraction effect of the pandemic on cancer care due to the overwhelmed system [23]. However, it is critical to educate cancer patients about the importance of adhering to physicians’ recommendations to minimize negative impacts on their treatment and outcome, and to provide psychological support and counseling if needed [24].

It is critical to increase the public knowledge of the risk of SARS-CoV-2 infection among cancer patients by advertising through trusted knowledge portals and advising patients to seek information from credible official sources to combat misconceptions and myths surrounding the disease [25,26].

Improving public knowledge is important as it will reflect on the attitude and behaviors of people and may reduce self-exposure to infection and transmission to others [14,19]. However, there should be awareness about the risks associated with avoiding seeking timely care. For example, in a study of patients with suspected breast lesions and breast cancer, there was a higher rate of procedure refusal and surgical procedure in the cohort with fear of COVID-19 [27]. In another study of endoscopic procedures, 29% of the patients selected did not show up for their procedures out of infection fear, and many of them were diagnosed with cancers [28].

Our study limitations are related to the method used and to the nature of the pandemic. Using electronic surveys may lead to selection biases of enrolling only those who have access to smartphones and can read and write; therefore we used other methods of enrolling more patients to capture a more representative sample of all cancer patients in the region. The study captured the status of patients’ knowledge and behavior at the study time, which may change with time as the pandemic evolves in terms of its severity and the knowledge about it gets better with time [20].

Although our study was conducted in the MENA region, the large sample size and representation of different healthcare systems in different countries with variable socioeconomic backgrounds make our findings relevant to different populations across the world. The concerns and fears, the impact of knowledge, variation in adherence to precautionary measures, avoidance of medical care, preference for virtual visitation and other factors have been reported in different populations. Thus lessons learned from this study could be of benefit to the global oncology community [29–33].

## Conclusion

Our study of a large number of cancer patients in the MENA region revealed the correlation of different variables with poor precautionary measures against COVID-19. Improving patients’ knowledge is a critical step to address this issue and other behaviors that may expose patients to additional risks such as avoiding medical care. Co-ordinated and well-designed educational interventions are needed for cancer patients and their caregivers.

**Summary points**

- This is a cross-sectional study utilizing a survey to assess the behavior and practices of patients with cancer in response to COVID-19 pandemic. The study included patients from 13 centers in six countries: Algeria, Egypt, Jordan, Kuwait, Morocco and Saudi Arabia.
- Between 21 April and 30 May 2020, a total of 3642 patients were enrolled in the study. The patients’ median age was 55 years (range: 14–97), 59.23% were female and 75.37% reported being married. Breast cancer was the most common malignancy reported and 83% of patients had received cancer therapy in the last 3 months.
- More than one-third of the participants either did not believe or did not know that an asymptomatic person can be a carrier of the virus. About three-quarters of patients did believe that infection is more severe in immunocompromised patients.
- A majority of the patients reported being either very worried (46.81%) or slightly worried (38%) about contracting the infection.
- 10–30% of the participants did not adhere to precautionary measures such as hand washing, wearing masks in public or maintaining social distancing.
- About 21% of patients admitted that they would not attend a previously scheduled medical appointment at this time; others canceled their appointments (16%) or treatment sessions (12.66%).
- A multivariate regression analysis on the predictors of poor precautionary practices revealed that significant variables are: male sex, divorced or widowed, disease in remission, canceling medical appointment or treatment session and having poor knowledge about COVID-19.
